# Chronotype and FibroScan-Derived Liver Markers in Adults with Obesity: Preliminary Evidence from a Cross-Sectional Study

**DOI:** 10.3390/nu18142342

**Published:** 2026-07-16

**Authors:** Nicole Cerabino, Davide Guido, Leonilde Bonfrate, Endrit Shahini, Vincenza Di Stasi, Martina Di Chito, Maria Noemy Pastore, Sergio Coletta, Anna Ancona, Gianluigi Giannelli, Giovanni De Pergola

**Affiliations:** 1 Center of Nutrition for the Research and the Care of Obesity and Metabolic Diseases, National Institute of Gastroenterology IRCCS “Saverio de Bellis”, 70013 Castellana Grotte, Bari, Italy; nicole.cerabino@irccsdebellis.it (N.C.); leonilde.bonfrate@irccsdebellis.it (L.B.); vincenza.distasi@irccsdebellis.it (V.D.S.); martina.dichito@irccsdebellis.it (M.D.C.); giovanni.depergola@irccsdebellis.it (G.D.P.); 2 Unit of Data Science, National Institute of Gastroenterology IRCCS “Saverio de Bellis”, 70013 Castellana Grotte, Bari, Italy; noemy.pastore@irccsdebellis.it; 3 Department of Human Sciences and Promotion of the Quality of Life, University San Raffaele, 00166 Rome, Italy; 4 Department of Gastroenterology, National Institute of Gastroenterology IRCCS “Saverio de Bellis”, 70013 Castellana Grotte, Bari, Italy; endrit.shahini@irccsdebellis.it; 5 Core Facility Biobank, National Institute of Gastroenterology IRCCS “Saverio de Bellis”, 70013 Castellana Grotte, Bari, Italy; sergio.coletta@irccsdebellis.it (S.C.); anna.ancona@irccsdebellis.it (A.A.); 6 Scientific Direction, National Institute of Gastroenterology IRCCS “Saverio de Bellis”, 70013 Castellana Grotte, Bari, Italy; gianluigi.giannelli@irccsdebellis.it

**Keywords:** circadian rhythm, liver steatosis, liver fibrosis, FibroScan, endocrine function, lifestyle factors

## Abstract

**Background/Objectives:** Chronotype describes individual preferences regarding the timing of sleep, food intake, and other daily activities and is commonly evaluated using the Morningness–Eveningness Questionnaire (MEQ). Individuals with a morning chronotype generally prefer earlier sleep–wake schedules, whereas those with an evening chronotype exhibit delayed circadian timing and achieve peak alertness later in the day. Previous evidence has linked evening chronotype to an elevated risk of obesity and metabolic dysfunction; however, its association with Metabolic Dysfunction-Associated Steatotic Liver Disease (MASLD), particularly with hepatic steatosis and fibrosis, has not yet been fully elucidated. **Methods:** This cross-sectional study included 119 medication-naïve adults, predominantly with obesity (mean age 41.5 ± 11.9 years; 58 men, 49%), recruited at the Nutrition Center of the National Institute of Gastroenterology “Saverio de Bellis.” Hepatic steatosis (controlled attenuation parameter [CAP] > 275 dB/m) and liver fibrosis (liver stiffness > 8.2 kPa) were assessed by transient elastography (FibroScan). **Results:** Hepatic steatosis was detected in 62% of participants (*n* = 74), whereas fibrosis was identified in 15% (*n* = 18). When chronotype was analyzed as a continuous variable, no significant correlations were observed with either CAP (r = 0.017, *p* = 0.852) or liver stiffness measurements (r = −0.045, *p* = 0.629). Although these associations did not reach statistical significance, the corresponding confidence intervals remained compatible with small-to-moderate effect sizes, particularly for fibrosis, reflecting the limited number of participants with fibrotic disease. **Conclusions:** In this sample, chronotype was not significantly associated with FibroScan-derived measures of hepatic steatosis or fibrosis. Associations with selected metabolic and behavioral characteristics were identified, although these findings require confirmation in adequately powered longitudinal investigations. To our knowledge, only a limited number of studies have explored these relationships using objective FibroScan-derived measurements in medication-naïve individuals. Consequently, the present findings should be considered hypothesis-generating and warrant validation in larger prospective cohorts.

## 1. Introduction

Daily physiological and behavioral rhythms follow the circadian timing system, which allows endogenous biological processes to remain coordinated with the external light–dark cycle. This temporal regulation is relevant to metabolic balance, since circadian rhythms influence energy handling, endocrine activity, and behavioral patterns linked to nutrition and sleep [1]. In this context, chronotype refers to the individual tendency to prefer earlier or later timing of daily activities, including sleeping, waking, and eating. According to the Morningness–Eveningness Questionnaire (MEQ), individuals can be positioned along a continuum extending from eveningness to morningness.

Along this continuum, morning-oriented individuals usually prefer earlier schedules, whereas evening-oriented individuals more often shift their peak alertness and performance toward the later hours of the day [2].

From a clinical perspective, evening chronotype has attracted increasing interest because it is often accompanied by a poorer alignment between biological timing and social demands. This mismatch may affect sleep habits, meal timing, and other lifestyle behaviors that are relevant to metabolic health. This condition, commonly referred to as chrono-disruption, has been associated with less favorable weight and glucose profiles as well as with cardiovascular disease [3,4]. Emerging evidence also suggests that evening chronotype may coexist with increased inflammatory activity within visceral adipose tissue, a recognized marker of metabolic dysfunction and cardiometabolic risk [5].

Although circadian dysregulation is increasingly recognized as a contributor to metabolic disease, the specific role of chronotype in liver fat accumulation and fibrotic progression has not yet been fully clarified. Metabolic Dysfunction-Associated Steatotic Liver Disease (MASLD) is defined by the accumulation of fat within the liver in individuals presenting metabolic risk factors such as obesity, diabetes mellitus, or hypertension [6]. While hepatic fat accumulation represents the early and most common expression of the disease, progression toward steatohepatitis and fibrosis identifies patients at greater long-term risk, including those exposed to cirrhosis-related complications and hepatocellular carcinoma [7]. Among liver-related features, fibrosis is especially important because it reflects a more advanced disease stage, has major prognostic implications, and is driven by several pathophysiological mechanisms, including insulin resistance, oxidative stress, and chronic inflammation [8,9].

Growing interest has recently focused on the potential relationship between chronotype and NAFLD/MASLD [10]. Several studies have reported that an evening chronotype may be associated with a less favorable liver phenotype, including a higher probability of fibrosis [11], and greater hepatic fat content [12,13]. However, the majority of these investigations have relied on liver histology, ultrasonographic assessments, or biochemical surrogate indices, whereas relatively few have employed quantitative FibroScan-derived measurements to evaluate hepatic steatosis and fibrosis [14].

The present exploratory cross-sectional study examined whether chronotype, assessed using the Morningness–Eveningness Questionnaire (MEQ), was associated with FibroScan-derived measures of hepatic steatosis and liver fibrosis in medication-naïve adults with overweight or obesity. The working hypothesis was that greater eveningness would be associated with higher controlled attenuation parameter (CAP) and liver stiffness measurement (LSM) values. At the same time, because the MEQ captures behavioral preference rather than objective circadian disruption, and because detailed data on sleep features and chrono-nutrition were not available, the analysis was conceived as exploratory and hypothesis-generating. Hepatic steatosis and fibrosis were quantified by transient elastography (FibroScan) in a sample of untreated adults with overweight or obesity. Beyond examining the direct associations between chronotype and FibroScan-derived liver outcomes, we also explored whether chronotype was related to behavioral and metabolic characteristics that may indirectly contribute to liver health. This broader analytical approach may provide further insight into the complex interplay between chronotype, metabolic dysfunction, and liver-related outcomes.

## 2. Materials and Methods

### 2.1. Study Design and Population

We performed a cross-sectional investigation at the Center of Nutrition for Research and Management of Obesity and Metabolic Disorders, National Institute of Gastroenterology “Saverio de Bellis”, Castellana Grotte (Bari, Italy). The protocol was prospectively recorded in the ClinicalTrials.gov registry (NCT05477212). Ethical clearance was granted by the Local Medical Ethics Committee of the National Institute of Gastroenterology and Research Hospital (protocols DDG-CE-502/2005 and DDG-CE-792/2014, approved on 20 May 2005 and 14 February 2014, respectively).

The present manuscript reports baseline cross-sectional findings derived from the registered clinical trial (NCT05477212_18 July 2022). In particular, chronotype, dietary variables, and FibroScan-derived liver measurements were examined as observational baseline features of the registered study. Study procedures complied with the ethical principles of the Declaration of Helsinki and with Good Clinical Practice guidelines.

Written informed consent was obtained from every participant before enrollment. Recruitment was carried out between February 2023 and July 2024. Inclusion criteria were age between 18 and 65 years, BMI ≥ 25 kg/m^2^, and absence of ongoing pharmacological treatment at study entry. Participants were excluded in the presence of previously diagnosed diabetes mellitus, cardiovascular disease, respiratory insufficiency, severe gastrointestinal disorders, chronic kidney disease (estimated glomerular filtration rate < 60 mL/min/1.73 m^2^), psychiatric illness, pregnancy or breastfeeding, eating disorders, chronic liver disease unrelated to metabolic dysfunction, alcohol intake above 30 g/day in men or 20 g/day in women, substance abuse, frailty, acute infectious or inflammatory conditions that could influence inflammatory markers, or rare inherited metabolic disorders such as porphyria, carnitine deficiency, carnitine palmitoyl transferase deficiency, carnitine–acylcarnitine translocase deficiency, pyruvate carboxylase deficiency, and disorders of mitochondrial fatty acid oxidation. At study entry, information on smoking and alcohol consumption was recorded together with demographic data, anthropometric assessment, and fasting blood sampling. Liver stiffness and steatosis were then evaluated by transient elastography (FibroScan®_Echosens, Paris, France) within one week of enrollment.

### 2.2. Anthropometric Parameters

All anthropometric measurements were obtained during morning visits following an overnight fasting period. Participants were evaluated wearing light clothing, barefoot, and after emptying the bladder. Body mass index (BMI) was calculated by dividing body weight (kg) by the square of height (m^2^) [15]. Waist circumference was obtained with the participant standing, placing the tape midway between the lower costal margin and the iliac crest, and expressed in centimeters. Arterial blood pressure was assessed after a resting period in the seated position using an automated sphygmomanometer (OMRON M6_ OMRON Healthcare Co., Ltd., Kyoto, Japan), and the mean value of three consecutive measurements was considered for analysis.

### 2.3. Morningness–Eveningness Questionnaire (MEQ)

Chronotype was evaluated with the Morningness–Eveningness Questionnaire (MEQ), using the validated Italian adaptation of the original Horne and Östberg instrument [16,17]. MEQ consists of 19 items that explore preferred sleep–wake timing, perceived efficiency at different times of day, and usual scheduling of daily activities. Individual scores position participants along a continuum, with lower values indicating greater evening preference and higher values indicating morning orientation. In the present analysis, MEQ values were treated as a continuous variable in order to preserve the full information content and optimize statistical power, rather than using predefined chronotype categories. The MEQ is widely adopted in clinical and research settings to characterize interindividual differences in circadian preference and their associations with physiological, behavioral, and cognitive measures.

### 2.4. Assessment of Liver Steatosis and Fibrosis

Liver fat accumulation and fibrosis were assessed through vibration-controlled transient elastography (FibroScan), a non-invasive imaging method extensively applied in subjects with metabolic risk conditions [18]. In this setting, compared with liver biopsy, FibroScan provides a convenient non-invasive approach for estimating hepatic fat content and tissue stiffness across a substantially larger liver volume, and is recommended by current guidelines as a first-line tool for the non-invasive staging of metabolic-associated steatotic liver disease [19].

Examinations were carried out by trained staff following standard operating procedures and international recommendations. The M or XL probe was chosen according to body habitus, with the XL probe preferentially used in participants with obesity. Examinations were considered technically acceptable when at least ten valid acquisitions were obtained, the success rate reached or exceeded 60%, and the interquartile range/median ratio remained below 30%, consistent with EASL recommendations [19].

Hepatic steatosis was considered present when CAP values exceeded 275 dB/m [19]. Controlled attenuation parameter (CAP), measured at 3.5 MHz during vibration-controlled transient elastography (VCTE), was used to estimate hepatic fat content. Liver stiffness measurements (LSM) obtained by VCTE served as an indirect estimate of hepatic fibrosis through assessment of liver tissue stiffness. According to current recommendations, liver fibrosis was evaluated using a rule-out threshold of 8 kPa and a rule-in threshold of 12 kPa for advanced (grade 3) fibrosis [19]. For binary analyses, significant liver fibrosis was defined as LSM > 8 kPa.

### 2.5. Laboratory Tests

Fasting venous blood samples were obtained in the early morning (08:00–09:00 h) following overnight fasting. Serum and plasma samples were analyzed for fasting plasma glucose (FPG), fasting insulin, triglycerides, total cholesterol, low-density lipoprotein cholesterol (LDL-C), high-density lipoprotein cholesterol (HDL-C), aspartate aminotransferase (AST), alanine aminotransferase (ALT), γ-glutamyl transferase (GGT), uric acid, creatinine, ferritin, thyroid function parameters, and 25-hydroxyvitamin D concentrations using the COBAS 8000 automated analyzer (ROCHE Diagnostic SPA, Monza, Italy).

HbA1c concentrations were determined using the Capillarys 3 OCTA capillary electrophoresis platform (Sebia Italia S.r.l., Bagno a Ripoli, Florence, Italy).

Insulin resistance was assessed through the HOMA-IR index [20], calculated according to the standard formula:

HOMA-IR = [FPG (mg/dL) × fasting insulin (μIU/mL)]/405.

Physical activity was evaluated using the International Physical Activity Questionnaire (IPAQ) [21], whereas adherence to the Mediterranean diet was assessed through the PREDIMED questionnaire [22]. Chronotype was evaluated using the self-administered Morningness–Eveningness Questionnaire (MEQ). All questionnaires were completed on the same day as the FibroScan examination.

### 2.6. Study Outcomes

The primary outcome was the hepatic steatosis evaluated through FibroScan-derived CAP values. The primary endpoint was the correlation coefficient (r) describing the association between CAP and chronotype score. The secondary outcome was the liver stiffness measurements (LSM), obtained by FibroScan, and the relative correlation coefficient (r) with the chronotype score was the secondary endpoint.

### 2.7. Exposure Variables and Confounders

Chronotype score was considered the primary exposure variable. In line with existing evidence, age, sex, and HOMA-IR were identified a priori as essential confounding variables. BMI and waist circumference were additionally included to account for overall and abdominal adiposity. Thus, the fully adjusted models included age, sex, HOMA-IR, BMI, and waist circumference. Smoking status, physical activity, and adherence to the Mediterranean diet were not included in the adjustment models because they were conceptualized as potential behavioral correlates or possible mediators of chronotype rather than true confounders.

### 2.8. Statistical Analyses

Quantitative variables are reported as mean values with corresponding standard deviations, whereas categorical data are expressed as absolute frequencies and percentages. Preliminary univariate associations between FibroScan-derived parameters (CAP and LSM), chronotype, and clinical variables were evaluated using Pearson or Spearman correlation coefficients, as appropriate.

Associations between chronotype and liver-related outcomes were further examined using multivariable linear regression models adjusted for age, sex, HOMA-IR, BMI, and waist circumference. Corresponding unadjusted regression models were also fitted to estimate crude regression coefficients (β). Normality of data distributions was assessed by the Shapiro–Wilk test, and parametric methods were applied when assumptions were met. Given the exploratory nature of the study, no formal adjustment for multiple comparisons was implemented; therefore, all findings should be interpreted with appropriate caution. To investigate the associations between chronotype and dichotomous liver outcomes, Firth’s penalized logistic regression models for small sample sizes [23] were fitted using binary definitions of hepatic steatosis (CAP > 275 dB/m) and liver fibrosis (LSM > 8 kPa). This modeling strategy is recommended when the events-per-covariate (EPC) ratio is below 10, as conventional logistic regression may yield biased parameter estimates under these circumstances [24].

Adjusted odds ratios (ORs) and corresponding 95% confidence intervals (95%CI) were estimated after controlling for age, sex, HOMA-IR, BMI, and waist circumference.

For regression analyses, MEQ scores were rescaled by dividing the original values by 10 to improve interpretability of the regression coefficients. Consequently, regression estimates represent the expected change in the outcome associated with each 10-point increase in chronotype score, thereby facilitating comparison with the observed standard deviation.

Potential confounders were selected a priori according to their recognized demographic, metabolic, and clinical relevance within the hypothesized pathways linking chronotype and liver outcomes. Multicollinearity among explanatory variables was assessed using variance inflation factors (VIF), and variables with VIF values exceeding 5 were excluded from the final multivariable models.

The correlation coefficient (r) was used to quantify the strength of the unadjusted linear relationship between two continuous variables and represents one of the most widely accepted measures of effect size [25]. Correlation coefficients range from 0 (no linear association) to ±1 (perfect positive or negative linear association). According to Cohen’s recommendations, absolute values of approximately 0.10, 0.30, and 0.50 correspond to small, medium, and large effect sizes, respectively [26,27].

Statistical significance was defined as a two-sided *p* value < 0.05, and 95% confidence intervals were also reported. Because of the exploratory nature of the study and the absence of multiplicity correction, results with *p* values between 0.05 and 0.10 were considered only weakly suggestive and interpreted with considerable caution. Consistent with current methodological recommendations, the interpretation of statistically non-significant findings relied primarily on the magnitude of effect size estimates and their corresponding confidence intervals.

All analyses were performed using R software (v 4.3.3) [28] and its packages finalfit (v 1.1.0) [29], ggplot2 (v 4.0.3) [30], Hmisc (v 5.2.5) [31], brglm (v 0.7.3) [32], brglm2 (v 1.1.0) [33], and pwrss (v 1.2.0) [34]. Missing data represented less than 5% of the dataset and were imputed using the Random Forest algorithm implemented in the missForest (v 1.6.1) package [35]. This approach has demonstrated superior performance compared with several alternative imputation methods, particularly in datasets characterized by complex interactions and nonlinear relationships, while maintaining high computational efficiency and suitability for high-dimensional data [36].

## 3. Results

### 3.1. Descriptive Statistics

Table 1 summarizes descriptive statistics. In total, 119 participants (41.5 ± 11.90 yrs; 58 men, 48.73%) were analyzed. A total of 23 subjects were current smokers (19%). Seventy-four individuals (62%) had liver steatosis, comprising 43 men (74.14%) and 31 women (50.82%); meanwhile, 18 participants (15.12%) had liver fibrosis, including 10 men (17.24%) and 8 women (13.11%). The mean chronotype score was 56.34 ± 8.88 (MEQ units). According to established cut-off values, 46 participants (38.7%) were classified as evening type subjects (chronotype ≤ 52), 51 (42.9%) with neither type (53 ≤ chronotype ≤ 64), and 22 (18.5%) morning-type participants (chronotype ≥ 65), according to established cut-off values [32]. The mean CAP and LSM values were 290.63 ± 54.53 dB/m and 6.45 ± 4.25 kPa, respectively. The mean IPAQ score was 1752.14 ± 1983.37, suggesting generally moderate-to-high levels of physical activity. Categories obtained by MET threshold values (low: 0 < MET < 600; moderate: 600 ≤ MET < 3000; high: ≥3000) provide the following percentage frequencies: 18 (15.1%), 84 (70.6%), and 17 (14.3).

Based on anthropometric measurements, 116 participants had obesity (55 males) and 3 had overweight (males). Mean BMI and waist circumference were 37.28 ± 5.81 kg/m^2^ and 113.76 ± 13.53 cm, respectively. None had a previous diagnosis of cardiovascular disease. Excess adiposity was accompanied by several metabolic abnormalities, including mild elevation of both systolic and diastolic arterial pressure (132.44 ± 11.19 and 80.94 ± 8.74 mmHg, respectively). A total of 11 subjects (9.2%) had previously undiagnosed type 2 diabetes (T2D). Type 2 diabetes was identified during biochemical screening at enrollment and this did not preclude their inclusion in the statistical analyses; all subjects were naïve to drug treatment and were therefore included in the analyses. Baseline FPG was 95.82 ± 13.77 mg/dL, HbA1c was 5.57 ± 0.48%, and fasting plasma insulin was 22.53 ± 16.35 IU/mL. The mean HOMA-IR value was 5.52 ± 4.74, indicating marked insulin resistance (n.v. < 2.5). Apart from slightly lower-than-normal 25-hydroxyvitamin D concentrations and modest elevations in total cholesterol and LDL cholesterol, the remaining laboratory parameters were within the normal range.

To visualize the joint distribution between chronotype levels and FibroScan measurements, scatter plots (with regression lines) and contour plots were also extracted (Figure S1 in Supplementary Materials).

### 3.2. Exploratory Correlation Analysis

CAP values satisfied the assumptions for parametric analysis (*p* = 0.374) and were therefore examined using Pearson’s correlation coefficients. In contrast, LSM did not follow a normal distribution (*p* < 0.001), and correlations involving LSM were assessed using Spearman’s rank coefficients. Table 2 and the heatmaps in Figure S2 (see Supplementary Materials) present the results of the exploratory correlation analysis between FibroScan measurements and chronotype levels. No significant correlations were observed between chronotype and either CAP (r = 0.017, *p* = 0.852, 95% CI = −0.163; 0.197) or LSM (r = −0.045, *p* = 0.629, 95% CI = −0.225; 0.131) in this sample. Notably, there was a positive correlation between CAP and LSM (r = 0.276, *p* = 0.002, 95% CI = 0.103; 0.424). Table 2 also presents the Pearson correlations between chronotype and other clinical variables in this sample. Analyses showed small inverse correlations between chronotype and TSH (r = −0.237, *p* = 0.009, 95% CI= −0.402; -0.059) and FT3 (r = −0.213, *p* = 0.019, 95% CI= −0.365; −0.042), and small positive correlations with vitamin D levels (r = 0.223, *p* = 0.015, 95% CI = 0.045; 0.388) and Mediterranean diet adherence (r = 0.189, *p* = 0.040, 95% CI = −0.006; 0.369). Given the number of exploratory correlations examined and the absence of multiplicity adjustment, these findings should be considered preliminary and hypothesis-generating.

Moreover, regarding the associations between FibroScan markers and other clinical and laboratory variables, we observed a significant direct correlation between CAP values and parameters of excess weight (BMI and WC), insulin resistance, glucose control, and liver cytotoxicity (AST and ALT). Similar associations were observed for liver stiffness. Correlations between CAP and glucose control markers varied between 0.165 and 0.370, while correlations with LSM were slightly higher between 0.120 and 0.322. Similarly, the reported correlations between FibroScan markers and liver cytotoxicity ranged from 0.154 to 0.395.

### 3.3. Regression Analysis

Table 3 illustrates the results of multiple regression analyses, including the associations between chronotype and FibroScan measurements controlled by sex, age, HOMA-IR, BMI and waist circumference. No association was observed for both CAP (β = 0.052, *p* = 0.992, 95% CI = −10.632; 10.736) and LSM (β = 0.024, *p* = 0.949, 95% CI = −0.725; 0.774). Notably, simple linear models also revealed no significant associations: CAP (β = 0.950, *p* = 0.867, 95% CI = −10.292; 12.192); LSM (β = −0.102, *p* = 0.816, 95% CI= −0.977; 0.772). Table 3 also shows Firth’s logistic regression models. No significant association between chronotype and steatosis and fibrosis was observed in the ordinary model (steatosis, OR = 1.058, *p* = 0.789, 95% CI= 0.696; 1.608; fibrosis, OR = 1.532, *p* = 0.184, 95% CI = 0.816; 2.879) or in the multiple models (steatosis, OR = 0.999, *p* = 0.997, 95% CI = 0.610; 1.638; fibrosis, OR = 1.568, *p* = 0.181, 95% CI = 0.810; 3.036). Moreover, a regression-based sensitivity analysis was carried out by considering the chronotype regressor as categorized (trichotomized) variable (see the Supplementary Materials). No covariate was excluded from the adjustment set because all variance inflation factors (VIFs) were <5, indicating no relevant multicollinearity.

## 4. Discussion

In the present sample, chronotype was not clearly associated with FibroScan-derived indices of hepatic steatosis or fibrosis. Nevertheless, the confidence intervals remained compatible with small-to-moderate effect sizes, particularly for liver fibrosis, so the possibility of clinically relevant associations cannot be definitively dismissed. The relatively small number of participants presenting liver fibrosis (*n* = 18) limited the precision of the estimates related to this outcome and requires cautious interpretation of the corresponding findings. While the study did not support the initial hypothesis, it provides preliminary evidence from a cohort of untreated individuals that may contribute to the design of future prospective research. It should also be recognized that the Morningness–Eveningness Questionnaire (MEQ) assesses individual preferences for the timing of sleep and daily activities rather than directly measuring circadian disruption. Consequently, these findings should not be considered sufficient to rule out a possible involvement of circadian dysregulation in the development or progression of MASLD. Furthermore, because of the cross-sectional design, no conclusions can be drawn regarding causality, intermediary mechanisms, or the temporal relationships linking chronotype with liver-related outcomes. Consequently, biological mechanisms potentially linking chronotype to hepatic health remain hypothetical and require confirmation through prospective studies.

It is also important to distinguish chronotype from other aspects of circadian misalignment. Variables such as social jetlag, habitual sleep duration and quality, exposure to shift work, and the timing and distribution of food intake may provide more proximal indicators of circadian disruption. These dimensions were not captured in the present study and should be incorporated into future work to obtain a more comprehensive picture of circadian influences on MASLD and related liver outcomes.

### 4.1. Study Strengths

Several aspects enhance the value of the present study. Evidence regarding the relationship between chronotype and liver health in untreated individuals with overweight or obesity remains scarce, making this population of particular interest. By restricting enrollment to participants not undergoing pharmacological treatment, potential confounding effects related to medication use were reduced, thereby improving the interpretability of the observed findings. Focusing on medication-naïve participants reduced the likelihood that drugs could modify metabolic variables or liver-related markers, thereby simplifying interpretation of the associations.

A second strength is the use of FibroScan as the main tool for assessing hepatic steatosis and fibrosis. This non-invasive technique, applied according to international recommendations, offers quantitative and reproducible measurements and is widely integrated into clinical pathways for liver disease evaluation. Its use increases the robustness and clinical relevance of the liver outcomes considered in the analysis.

The study also benefited from an extensive phenotypic characterization of the participants. Anthropometric indices, clinical and biochemical parameters, hormonal profiles, and dietary habits were collected within the same protocol, providing a broad view of metabolic status. The inclusion of a validated instrument to assess adherence to the Mediterranean diet (PREDIMED) allowed the findings to be interpreted within a wider lifestyle and behavioral contest. Taken together, these elements support the internal validity of the data and offer a solid basis for the development of future longitudinal studies aimed at clarifying how chronotype, metabolic dysfunction, and liver disease may interact over time.

### 4.2. Limitations of the Study

Despite these strengths, several limitations should be acknowledged. The primary limitation is the cross-sectional design, which prevents the establishment of causal relationships between chronotype and hepatic alterations. Therefore, the observed findings should be considered exploratory and hypothesis-generating. Although associations between chronotype and selected behavioral or metabolic characteristics were identified, these results do not demonstrate that chronotype directly contributes to the development of these alterations. Furthermore, correlation analyses quantify the magnitude of linear associations but cannot determine temporal relationships or mediation mechanisms. Longitudinal studies are therefore required to clarify the potential long-term impact of chronotype on metabolic and liver health.

Given the exploratory nature of the investigation, no correction for multiple comparisons was applied; therefore, secondary findings should be interpreted with caution. Additionally, the limited number of participants with fibrosis reduced the precision of fibrosis-related estimates and may have limited the ability to identify modest associations.

Another limitation concerns the assessment of chronotype using the Morningness–Eveningness Questionnaire (MEQ). Although the MEQ is a validated instrument widely used in chronobiological research, it remains a subjective measure and cannot substitute for objective assessments of circadian timing, such as actigraphy-based evaluations or hormonal markers. Future studies incorporating objective measures of circadian function would provide a more comprehensive characterization of biological rhythmicity.

An additional limitation is the lack of detailed information regarding chrono-nutrition and sleep-related variables. Specifically, sleep duration and quality, social jetlag, shift work exposure, habitual bedtimes and wake times, meal timing, eating window duration, eating frequency, and circadian distribution of energy intake were not evaluated. These factors represent important determinants of chrono-nutritional behavior and may influence both chronotype and liver-related outcomes, potentially acting as mediators or confounders in the relationship between chronotype and MASLD. Consequently, the absence of detailed sleep and dietary timing data limits the ability to determine whether potential associations with MASLD are attributable to chronotype itself or to associated behavioral patterns. Future longitudinal studies integrating objective circadian measurements and comprehensive chrono-nutrition assessments are warranted.

The study population was relatively small and mainly composed of younger adults with limited clinical variability. Moreover, participants were recruited from an obesity referral center, which may reduce generalizability and introduce selection bias. The limited sample size, particularly the small number of individuals with fibrosis, decreased statistical power and may have prevented the detection of weaker associations. Although multivariable adjustments were performed, residual confounding cannot be excluded.

Finally, the absence of a healthy control group represents an additional limitation. Including a comparison group would have facilitated a clearer evaluation of differences between individuals with and without liver alterations. Furthermore, the single-center design, relatively young population, limited fibrosis subgroup, and absence of external validation restrict the generalizability of the findings.

### 4.3. Implications for Clinical Practice

Although no significant associations between chronotype and liver-related outcomes were identified, exploratory analyses suggested possible relationships between chronotype and selected behavioral and endocrine parameters. However, these secondary analyses were conducted without adjustment for multiple comparisons and were based on a relatively limited sample size. Therefore, associations involving thyroid-stimulating hormone (TSH), free triiodothyronine (FT3), vitamin D levels, and PREDIMED scores should be considered preliminary and hypothesis-generating. Confirmation in larger, adequately powered longitudinal cohorts is required before clinical implications can be established.

## 5. Conclusions

In this sample of medication-naïve adults, predominantly with obesity, no statistically significant association was detected between chronotype and FibroScan-derived measures of hepatic steatosis or fibrosis. Because the confidence intervals remain compatible with small-to-moderate effects, particularly for fibrosis, clinically relevant associations cannot be excluded. Future adequately powered longitudinal studies incorporating objective measures of circadian disruption and chrono-nutrition behaviors are needed to clarify whether chronotype contributes to MASLD development or progression.

## Figures and Tables

**Table 1:** Background characteristics of the study sample.

Variables (*n* = 119)	Mean ± SD or *n* (%)
*Ultrasonographic measures of liver steatosis and fibrosis*	
FibroScan CAP (dB/m)	290.63 ± 54.53
FibroScan LSM (kPa)	6.45 ± 4.25
Steatosis * (yes/no; *n* and %)	74/45 (62/38)
Fibrosis * (yes/no; *n* and %)	18/101 (15/85)
*Variable of exposure*	
Chronotype score (u)	56.34 ± 8.88
Chronotype (Eveningness/Neither type/Morningness)	46/51/22 (38.7/42.9/18.5)
*Hallmarks of glucose control and insulin resistance*	
FPG (mg/dL)	95.82 ± 13.77
HbA1c (%)	5.57 ± 0.48
Fasting serum insulin (U/mL)	22.53 ± 16.35
HOMA-IR (score)	5.52 ± 4.74
Diabetes (yes/no)	11/108 (9.2/90.8)
*Demographic and lifestyle characteristics*	
Age (yrs)	41.50 ± 11.90
Sex (male/female; *n* and %)	58/61 (49/51)
Current smokers (yes/no; *n* and %)	23/96 (19/81)
PREDIMED score (adherence to Mediterranean diet) (u)	8.35 ± 1.95
IPAQ (MET)	1752.14 ± 1983.37
IPAQ (low/moderate/high)	18/84/17 (15.1/70.6/14.3)
*Anthropometric and clinical parameters*	
BMI (kg/m^2^)	37.28 ± 5.81
Waist circumference (cm)	113.76 ± 13.53
Systolic blood pressure (mmHg)	132.44 ± 11.19
Diastolic blood pressure (mmHg)	80.94 ± 8.74
*Laboratory tests*	
Triglycerides (mg/dL)	119.19 ± 68.53
HDL cholesterol (mg/dL)	48.91 ± 11.14
LDL cholesterol (mg/dL)	124.86 ± 25.93
Total cholesterol (mg/dL)	189.01 ± 29.85
TSH (μmU/mL)	2.27 ± 2.41
FT3 (pg/mL)	3.96 ± 6.40
FT4 (ng/dL)	11.75 ± 1.64
25-hydroxyvitamin D (ng/mL)	20.06 ± 6.82
Uric acid (mg/dL)	5.52 ± 1.41
Creatinine (mg/dL)	0.81 ± 0.14
AST (U/L)	24.13 ± 18.35
ALT (U/L)	34.47 ± 30.02
GGT (U/L)	35.05 ± 36.24
High-sensitive C-reactive protein (mg/dL)	0.50 ± 0.78
Ferritin (ng/mL)	179.67 ± 185.08

**Abbreviations:** *n*: sample size; u: unit; BMI: Body Mass Index; CAP, Controlled Attenuation Parameter; LSM, Liver Stiffness Measurement; FPG, Fasting Plasma Glucose; HbA1c, Glycated hemoglobin; HOMA-IR, Homeostasis Model Assessment–Insulin Resistance; IPAQ, International Physical Activity Questionnaire; HDL, High-Density Lipoprotein; LDL, Low- Density Lipoprotein; TSH, Thyroid-Stimulating Hormone; FT4, Free Tetraiodothyronine; FT3, Free Triiodothyronine; AST, Aspartate Amino Transferase; ALT, Alanine Amino Transferase; GGT, γ-Glutamyl Transferase; u, Standard Unit. * Steatosis and fibrosis were diagnosed in the presence of CAP > 275 dB/m and LSM > 8 kPa, respectively. Data are expressed as mean ± SD or *n* (%), as appropriate.

**Table 2:** Correlation coefficients describing the relationships of CAP, LSM, and chronotype with anthropometric, biochemical, and clinical variables in the study population (*n* = 119). Since these analyses were exploratory and no adjustment for multiple testing was performed, secondary associations should be viewed as preliminary findings requiring further confirmation.

Variables	CAP r (*p*-Value)	LSM r (*p*-Value)	Chronotype r (*p*-Value)
LSM	**0.276 (0.002)**	-	-
Chronotype	0.017 (0.852)	−0.045 (0.629)	-
FPG	*0.165 (0.073)*	0.120 (0.195)	*0.152 (0.099)*
HbA1c	**0.235 (0.010)**	0.129 (0.160)	0.057 (0.532)
Insulin	**0.370 (<0.001)**	**0.322 (<0.001)**	−0.048 (0.598)
HOMA-IR	**0.357 (<0.001)**	**0.308 (<0.001)**	−0.009 (0.919)
IPAQ	−0.131 (0.152)	**0.208 (0.022)**	−0.001 (0.988)
BMI	**0.345 (<0.001)**	**0.432 (<0.001)**	−0.120 (0.192)
WC	**0.416 (<0.001)**	**0.288 (0.001)**	−0.019 (0.831)
Systolic blood pressure	*0.164 (0.075)*	−0.047 (0.605)	0.110 (0.235)
Diastolic blood pressure	0.004 (0.965)	−0.124 (0.178)	0.089 (0.334)
Triglycerides	**0.302 (<0.001)**	0.029 (0.748)	0.036 (0.699)
HDL cholesterol	**−** **0.375 (<0.001)**	−0.043 (0.639)	0.067 (0.464)
LDL cholesterol	0.027 (0.768)	−0.029 (0.752)	0.080 (0.387)
Total cholesterol	0.004 (0.964)	−0.015 (0.874)	0.080 (0.382)
TSH	0.040 (0.663)	−0.082 (0.377)	**−** **0.237 (0.009)**
FT3	0.167 (0.887)	0.018 (0.844)	**−0.213 (0.019)**
FT4	0.086 (0.352)	0.024 (0.794)	−0.147 (0.110)
25-hydroxyvitamin D	**−** **0.213 (0.019)**	−*0.151 (0.099)*	**0.223 (0.015)**
Uric acid	**0.419 (<0.001)**	0.071 (0.437)	−0.044 (0.630)
Creatinine	0.142 (0.122)	0.018 (0.839)	−0.024 (0.794)
AST	**0.291 (0.001)**	*0.178 (0.052)*	0.021 (0.816)
ALT	**0.395 (<0.001)**	*0.154 (0.094)*	0.110 (0.232)
GGT	**0.395 (<0.001)**	0.045 (0.623)	0.080 (0.385)
Ferritin	**0.337 (<0.001)**	0.092 (0.318)	0.072 (0.434)
PREDIMED score	−0.109 (0.238)	−*0.157 (0.086)*	**0.189** (**0.040**)

**Abbreviations:** *n*: sample size; CAP, Controlled attenuation parameter; LSM, Liver stiffness measurement; FPG, Fasting Plasma Glucose; HbA1c, Glycated hemoglobin; HOMA-IR, Homeostasis model assessment–insulin resistance; IPAQ, International Physical Activity Questionnaire; BMI, Body mass index; WC, Waist circumference; HDL, High-density lipoprotein; LDL, Low-density lipoprotein; TSH, Thyroid-stimulating hormone; FT4, Free tetraiodothyronine; FT3, Free triiodothyronine; AST, Aspartate amino transferase; ALT, Alanine amino transferase; GGT, γ-glutamyl-transferase; r: Pearson correlation coefficient. In **bold**, *p* < 0.05; in *italics*, 0.05 ≤ *p* < 0.10.

**Table 3:** Regression models evaluating the relationship between chronotype and FibroScan-derived indicators of hepatic steatosis and fibrosis.

	β of Chronotype on FibroScan CAP Linear Model	β of Chronotype on FibroScan LSM Linear Model	OR of Chronotype on Steatosis—Logistic Models	OR of Chronotype on Fibrosis—Firth’s Logistic Models
**Ordinary model**	β = 0.950 *p* = 0.867 95% CI = −10.292; 12.192	β = −0.102 *p* = 0.816 95% CI = −0.977; 0.772	OR = 1.058 *p* = 0.789 95% CI = 0.696; 1.608	OR = 1.532 *p* = 0.184 95% CI = 0.816; 2.879
Multiple model *	β = 0.052 *p* = 0.992 95% CI = −10.632; 10.736	β = 0.024 *p* = 0.949 95% CI = −0.725; 0.774	OR = 0.999 *p* = 0.997 95% CI = 0.610; 1.638	OR = 1.568 *p* = 0.181 95% CI = 0.810; 3.036

**Abbreviations:** CAP, Controlled attenuation parameter; LSM, Liver stiffness measurement; β, linear regression coefficient, i.e., expected variation of the response variable in the model per 10-unit increase in chronotype. OR, Odds ratio, *p*, *p*-value. 95% CI: 95% confidence intervals. * Adjusted for sex, age, HOMA-IR, BMI and waist circumference. Summary of linear and logistic regression models assessing whether chronotype was associated with liver-related outcomes: the column presents the results returned by the models in terms of association measures (i.e., β or OR), the rows present the modeling nature.

## Data Availability

The data that support the findings of this study are not publicly available because they contain sensitive personal information. De-identified data may be made available from the corresponding author upon reasonable request and subject to institutional data protection policies.

## Supplementary Materials

The following supporting information can be downloaded at: https://www.mdpi.com/article/10.3390/nu18142342/s1, Figure S1. Scatter and contour plots of chronotype and ultrasonographic features of liver steatosis (CAP) and fibrosis (LSM); Figure S2. Heatmaps of the correlation analysis; Figure S3. Plots of the post hoc power analysis for Pearson correlation coefficients between chronotype and CAP and LSM; Table S1. Results of the sensitivity analysis. Refs. [37,38] are cited in Supplementary Materials file.

## Abbreviations

The following abbreviations are used in this manuscript:

MASLD	Metabolic Dysfunction-Associated Steatotic Liver Disease
MASH	Metabolic Steatohepatitis
CVD	Cardiovascular Diseases
BMI	Body Mass Index
WC	Waist Circumference
CAP	Controlled Attenuation Parameter
LSM	Liver Stiffness Measurement
FPG	Fasting Plasma Glucose
LDL-C	Low-Density Lipoprotein Cholesterol
HDL-C	High-Density Lipoprotein Cholesterol
AST	Aspartate-Amino Transferase
ALT	Alanine-Amino Transferase
GGT γ-	Glutamyl-Transferase
TSH	Thyroid-Stimulating Hormone
FT4	Free Tetraiodothyronine
FT3	Free Triiodothyronine
HbA1c	Glycated hemoglobin
HOMA-IR	Homeostasis Model Assessment of Insulin Resistance
IPAQ	International Physical Activity Questionnaire
PREDIMED	Prevention with Mediterranean Diet
MEQ	Morningness–Eveningness Questionnaire
