# Supplementary material for: Chronotype and FibroScan-Derived Liver Markers in Adults with Obesity: Preliminary Evidence from a Cross-Sectional Study
Source: Nutrients. 2026 Jul 16;18(14):2342. doi: 10.3390/nu18142342 (PMC13415371; doi:10.3390/nu18142342)
Supplement: Supplementary file 1 [file nutrients-18-02342-s001.zip › nutrients-4375172-supplementary.pdf]

Supplementary

Graphical analysis

Figure S1 shows the joint distribution between chronotype levels and FibroScan measurements, scatter plots (with regression lines) and contour plots. The left-side plots show the joint distributions between chronotype and CAP values, whereas the right-side plots show the distributions with LSM. The scatter plots report the x-y points of the patients (i.e., chronotype levels-CAP or chronotype levels-LSM), whereas contour plots are 2D kernel density estimations of the frequency of the x-y points.

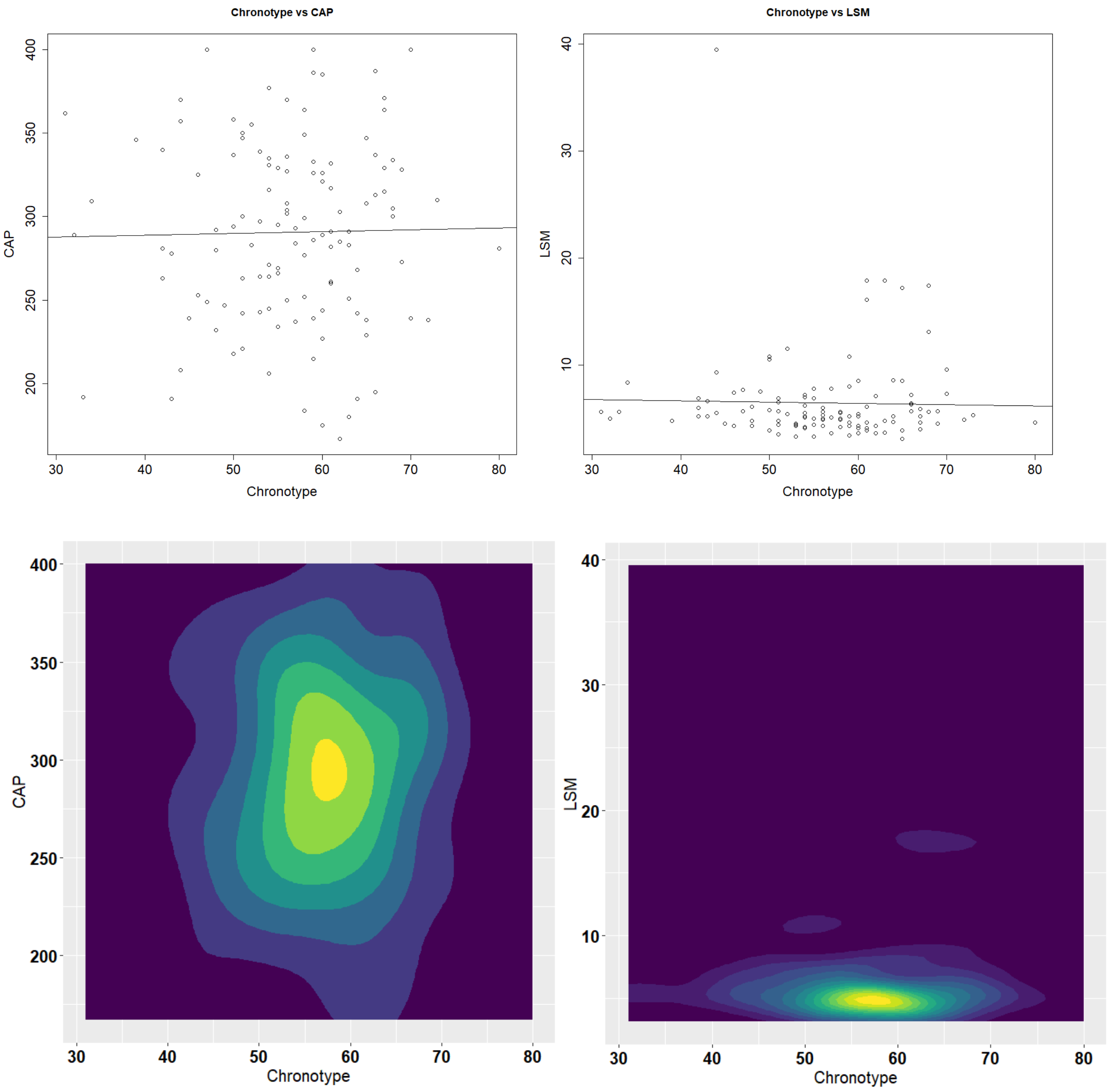

Figure S1. Scatter and contour plots of chronotype and ultrasonographic features of liver steatosis (CAP) and fibrosis (LSM).

Heatmap of the exploratory correlation analysis

Figure S2. Heatmaps of the correlation analysis.

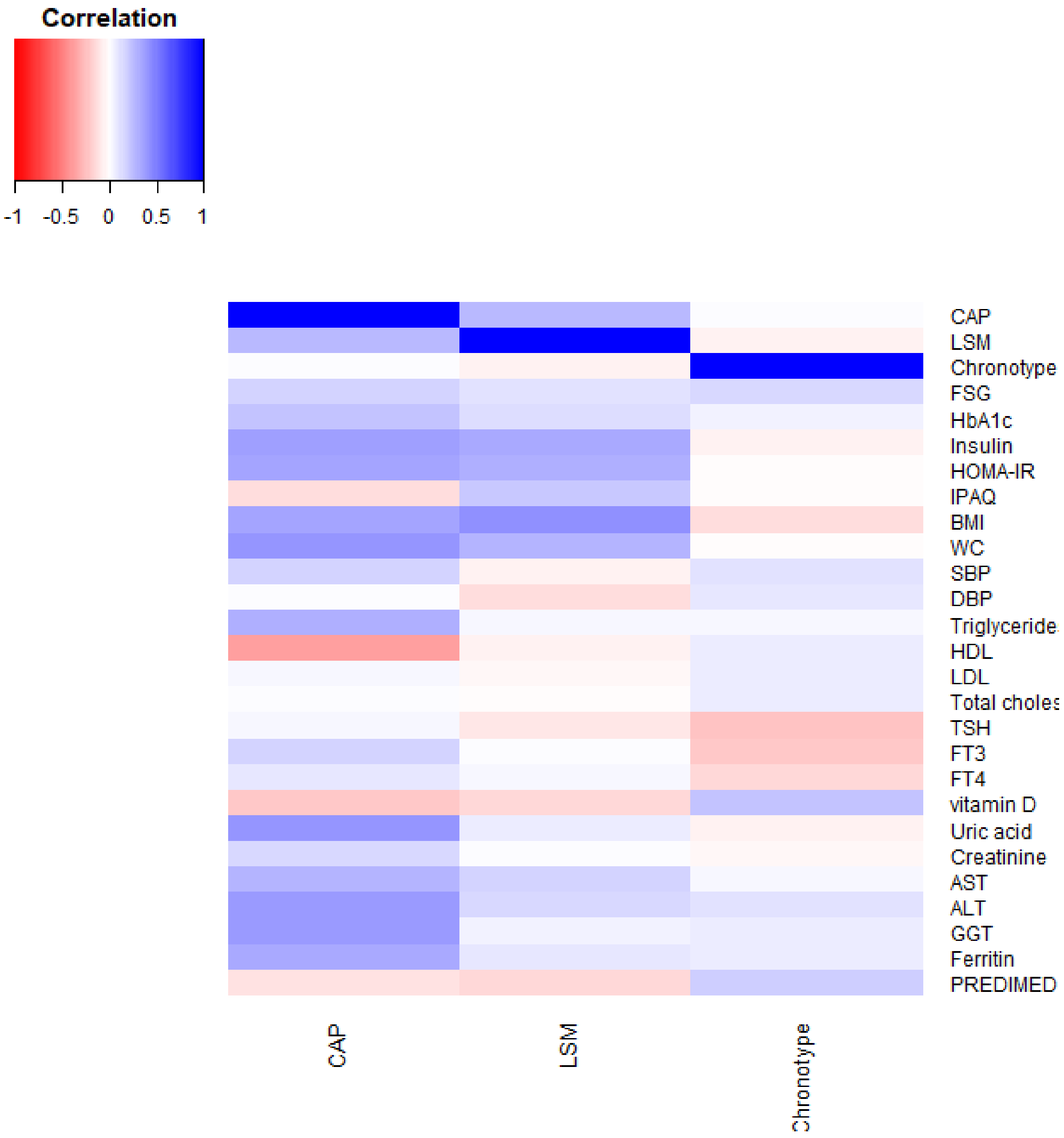

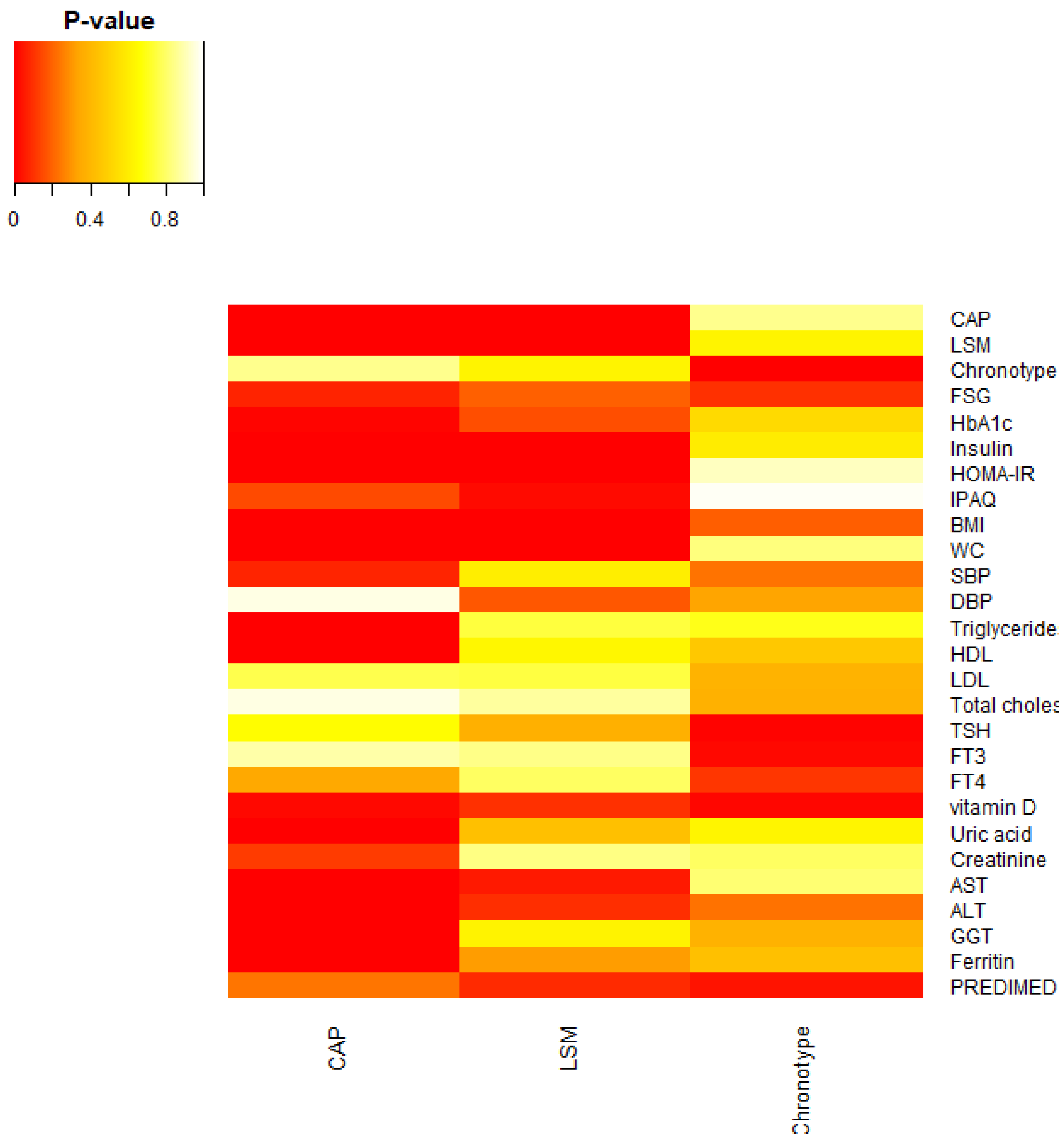
P-value
0
0.4
0.8
CAP
LSM
Chronotype
FSG
HbA1c
Insulin
HOMA-IR
IPAQ
BMI
WC
SBP
DBP
Triglyceride
HDL
LDL
Total choles
TSH
FT3
FT4
vitamin D
Uric acid
Creatinine
AST
ALT
GGT
Ferritin
PREDIMED
CAP
LSM
Chronotype

Sensitivity analysis

We conducted a sensitivity analysis on the statistical modelling by also considering the regressor chronotype as categorical (trichotomous) variable, by following the cutoff values by Taillard et al. [1]. Concerning that, CAP and LSM - and relative dichotomizations - were the response variables and ordinary and multiple linear and logistic regression models were fitted.

Table S1. Results of the sensitivity analysis

	**β of chronotype* on fibroscan CAP (linear model)**	**β of chronotype* on fibroscan LSM (linear model)**	**OR of chronotype* on steatosis (logistic model)**	**OR of chronotype* on fibrosis (logistic model)**
**Ordinary model**	$\beta_{neither}$ = - 6.494 p = 0.558 95%CI = - 28.405; 15.416 $\beta_{morningnness}$ = 15.895 p = 0.262 95%CI = - 12.036; 43.826	$\beta_{neither}$ = - 0.678 p = 0.435 95%CI = - 2.394; 1.037 $\beta_{morningnness}$ = 0.436 p = 0.694 95%CI = - 1.751; 2.623	$OR_{neither}$ = 1.090 p = 0.834 95%CI = 0.483; 2.458 $OR_{morningnness}$ = 1.876 p = 0.265 95%CI = 0.620; 5.675	$OR_{neither}$ = 1.304 p = 0.670 95%CI = 0.383; 4.436 $OR_{morningnness}$ = 2.411 p = 0.205 95%CI = 0.617; 9.419
**Multiple Model****	$\beta_{neither}$ = - 3.342 p = 0.760 95%CI = - 25.013; 18.327 $\beta_{morningnness}$ = 4.565 p = 0.731 95%CI = - 21.674; 30.805	$\beta_{neither}$ = 0.070 p = 0.926 95%CI = - 1.451; 1.593 $\beta_{morningnness}$ = 0.113 p = 0.902 95%CI = - 1.729; 1.957	$OR_{neither}$ = 1.062 p = 0.901 95%CI = 0.410; 2.749 $OR_{morningnness}$ = 1.313 p = 0.657 95%CI = 0.394; 4.379	$OR_{neither}$ = 1.667 p = 0.484 95%CI = 0.398; 6.988 $OR_{morningnness}$ = 1.992 p = 0.373 95%CI = 0.436; 9.097

Abbreviations. CAP, Controlled attenuation parameter; LSM, Liver stiffness measurement; b, linear regression coefficient, i.e., expected variation of the response variable in the model, in relation to the chronotype eveningness (reference category). OR, odds ratio. p, p-value. In **bold**, significant results (p<0.05), in *italics*, the trend (0.05<p<0.10); 95%CI: 95% confidence interval. *Eveningness is the reference category of the chronotype regressor; **Adjusted for sex, age, HOMA, BMI and waist circumference. Results of the regression analysis on the associations between Fibroscan measurements (outcomes) with chronotype categorized by following the cut-off values by Taillard et al. (2004) [1]:

i) chronotype ≤52 → evening-type subjects (i.e., eveningness);
ii) 53≤chronotype≤64 → neither type subjects;
iii) chronotype ≥65 → morning-type subjects (i.e., morningness);

the columns present the results returned by the models in terms of association measures (i.e., β or OR), the rows present the modelling nature.

Table S1 illustrates the results of the statistical modelling of the sensitivity analysis, including the associations between chronotype and FibroScan measurements controlled by sex, age, HOMA-IR, BMI and waist circumference. No association was observed for both CAP ($\beta_{neither}$ = -3.342, p = 0.760; $\beta_{morningnness}$ = 4.565, p = 0.731) and LSM ($\beta_{neither}$ = 0.070, p = 0.926; $\beta_{morningnness}$ = 0.113, p = 0.902). Notably, simple linear models also revealed no significant associations: CAP ($\beta_{neither}$ = -6.494, p = 0.558; $\beta_{morningnness}$ = 15.895, p = 0.262); LSM ($\beta_{neither}$ = -0.678, p = 0.435; $\beta_{morningnness}$ = 0.436, p = 0.694). Table also shows Firth's logistic regression models. No significant association between chronotype and steatosis and fibrosis values was observed in the ordinary model (steatosis, $OR_{neither}$ = 1.090, p = 0.834, $OR_{morningnness}$ = 1.876, p = 0.265; fibrosis, $OR_{neither}$ = 1.304, p = 0.901, $OR_{morningnness}$ = 2.411, p = 0.205) or in the multiple models (steatosis, $OR_{neither}$ = 1.062, p = 0.901; $OR_{morningnness}$ = 1.313, p = 0.657; fibrosis, $OR_{neither}$ = 1.667, p = 0.484; $OR_{morningnness}$ = 1.992, p = 0.373).

Post hoc power analysis

We conducted a post hoc power analysis using the Pearson correlation coefficients [2,3] among chronotype, CAP, and LSM. The two-sided Type I error level was set at 0.05. The post hoc power analysis is an estimate of the power of a test given the observed effect size and sample size. Post hoc power was explored descriptively to contextualize the observed effect sizes, although this approach has recognized limitations [4]. To determine all eligible sample size values, we conducted a simulation study to examine the performance of post hoc power analysis. We varied the power values (x-axis) and identified the corresponding sample sizes (y-axis) in relation to the observed effect size (Pearson correlation coefficients, r). However, it is important to note that post hoc power analysis has faced criticism, as well argued by Hoening & Heisey (2001) [5].

After calculations, post hoc analysis returned power values equal to 0.038 and 0.044 on the observed Pearson correlation coefficients of CAP ($r = 0.017$, $p = 0.852$) and LSM ($r = -0.024$, $p = 0.798$) with chronotype, respectively. Next, the simulation study determined the sample sizes based on power values, allowing the comparison with the power achieved in this study (Figure S3).

Figure S3. Plots of the post hoc power analysis for Pearson correlation coefficients between chronotype and CAP and LSM.

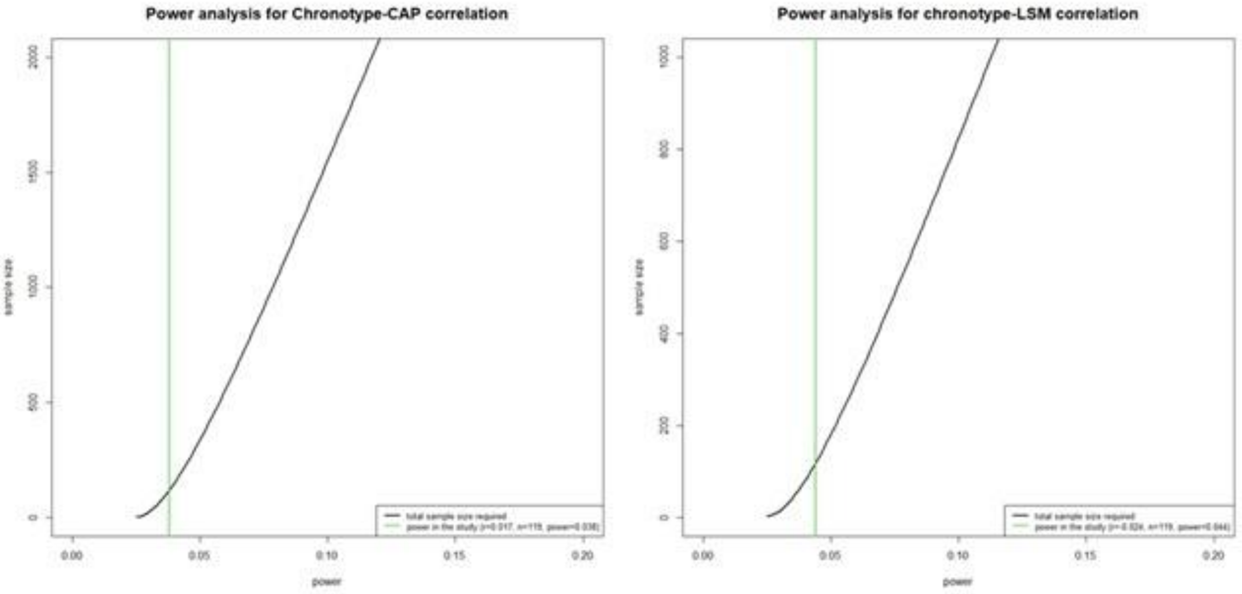

Abbreviations. CAP, Controlled attenuation parameter; LSM, Liver stiffness measurement.

R code of the post hoc power analysis

```
version #R version 4.3.3

#install.packages("pwrss") [6]
library(pwrss)
citation(package="pwrss")
```

```
######################################################################
###Post hoc power analysis: Pearson correlation chronotype-CAP###
######################################################################

#r: Linear correlation coefficient
#alpha
#power

n_pwrss<-NULL #n, total sample size required
pow <- seq(0.025,0.975,0.001)
for (i in pow){
n_pwrss<-c(n_pwrss,power.z.onecor(rho = 0.01725163,null.rho =
0,power = i,alpha = 0.05,alternative = "two.sided")$n)
}
n_pwrss
data.frame(pow,n_pwrss)



pwr.r.test(r=0.01725163,power=.8,sig.level=0.05,alternative="two.s
ided")
power.z.onecor(rho = 0.01725163,null.rho = 0,power = .8,alpha =
0.05,alternative = "two.sided")$n

pow[round(n_pwrss)==119] #0.038
data.frame(pow,n_pwrss) #valuta qualitativamente
(approssimativamente) da qui la potenza

tiff(filename="power.tiff", width=3500, height=2000, res=300)
par(mfrow=c(1,2))
plot(pow,n_pwrss, type="l", lwd=2, ylab="sample
size",xlab="power",main="Power analysis for Chronotype-CAP
correlation",xlim=c(0,0.2),ylim=c(0,2000),cex.main=1.5)
abline(v=0.038,lwd=2,col=3)
```

```
legend("bottomright",c("total sample size required","power in the
study (r=0.017, n=119,
power=0.038)"),lty=c(1,1),lwd=c(2,2),col=c(1,3),cex=0.8)
```

```
##################################################################
###Post hoc power analysis: Pearson correlation chronotype-LSM###
##################################################################

#r: Linear correlation coefficient
#alpha
#power


n_pwrss<-NULL #n, total sample size required
pow <- seq(0.025,0.975,0.001)
for (i in pow){
#n<-c(n,pwr.r.test(r=-
0.02367039,power=i,sig.level=0.05,alternative="two.sided")$n)
n_pwrss<-c(n_pwrss,power.z.onecor(rho = -0.02367039,null.rho =
0,power = i,alpha = 0.05,alternative = "two.sided")$n)
}
n_pwrss

data.frame(pow,n_pwrss)

pwr.r.test(r=-
0.02367039,power=.8,sig.level=0.05,alternative="two.sided")
power.z.onecor(rho = -0.02367039,null.rho = 0,power = 0.8,alpha =
0.05,alternative = "two.sided")$n

pow[round(n_pwrss)==119] #0.044
data.frame(pow,n_pwrss) #valuta qualitativamente
(approssimativamente) da qui la potenza


plot(pow,n_pwrss, type="l", lwd=2, ylab="sample
size",xlab="power",main="Power analysis for chronotype-LSM
correlation",xlim=c(0,0.2),ylim=c(0,1000),cex.main=1.5)
```

```
abline(v=pow[round(n_pwrss)==119],lwd=2,col=3)

legend("bottomright",c("total sample size required","power in the study (r=-0.024, n=119,
power=0.044)"),lty=c(1,1),lwd=c(2,2),col=c(1,3),cex=0.8)

dev.off()
```

References

[1] Jacques Taillard, Pierre Philip, Jean-François Chastang, and Bernard Bioulac, (2004). Validation of Horne and Ostberg Morningness-Eveningness Questionnaire in a Middle-Aged Population of French Workers. JOURNAL OF BIOLOGICAL RHYTHMS, Vol. 19 No. 1, February 2004 76-86. DOI: 10.1177/0748730403259849, Sage Publications.

[2] Cohen, J. Statistical Power Analysis for the Behavioral Sciences, 2nd ed.; Academic Press: New York, NY, USA, 1988. 525

[3] Cohen, J. Things I have learned (so far). *Am. Psychol.* **1990**, *45*, 1304–1312.

[4] Crespi, C.M. Power and Sample Size in R; Chapman & Hall: Boca Raton, FL, USA, **2020**; ISBN 9781138591622

[5] Hoening, J.M.; Heisey, D.M. The Abuse of Power: The Pervasive Fallacy of Power Calculations for Data Analysis. *Am. Stat.* **2001**, *55*, 19–24.

[6] Bulus, M.; Jentschke, S. pwrss: Statistical Power, Sample Size, and Detectable Effect Calculations. R Package version 1.2.0. 2026. Available online: https://cran.r-project.org/web/packages/pwrss/index.html (accessed on 20 April 2026).
